# Single molecule infrared spectroscopy in the gas phase

**DOI:** 10.1038/s41586-023-06351-7

**Published:** 2023-06-28

**Authors:** Aaron Calvin, Scott Eierman, Zeyun Peng, Merrell Brzeczek, Lincoln Satterthwaite, David Patterson

**Affiliations:** 1 Department of Physics, University of California, Santa Barbara, CA USA; 2 Department of Chemistry and Biochemistry, University of California, Santa Barbara, CA USA

**Keywords:** Chemical physics, Atomic and molecular physics, Physical chemistry

## Abstract

Spectroscopy is a key analytical tool that provides valuable insight into molecular structure and is widely used to identify chemical samples. Tagging spectroscopy is a form of action spectroscopy in which the absorption of a single photon by a molecular ion is detected via the loss of a weakly attached, inert ‘tag’ particle (for example, He, Ne, N_2_)^1–3^. The absorption spectrum is derived from the tag loss rate as a function of incident radiation frequency. So far, all spectroscopy of gas phase polyatomic molecules has been restricted to large molecular ensembles, thus complicating spectral interpretation by the presence of multiple chemical and isomeric species. Here we present a novel tagging spectroscopic scheme to analyse the purest possible sample: a single gas phase molecule. We demonstrate this technique with the measurement of the infrared spectrum of a single gas phase tropylium (C_7_H_7_^+^) molecular ion. The high sensitivity of our method revealed spectral features not previously observed using traditional tagging methods^4^. Our approach, in principle, enables analysis of multicomponent mixtures by identifying constituent molecules one at a time. Single molecule sensitivity extends action spectroscopy to rare samples, such as those of extraterrestrial origin^5,6^, or to reactive reaction intermediates formed at number densities that are too low for traditional action methods.

## Abstract (web summary)

Using tagging spectroscopy, the infrared spectrum of a single organic molecule in the gas phase has been successfully measured.

## Main

Single molecule spectroscopy in the condensed phase has been a fruitful area of research for decades^7,8^, but distortions from interaction-induced effects are unavoidable in condensed phase spectra. Gas phase single molecule spectroscopy has so far been limited to photodissociation and quantum logic measurements of diatomic molecular ions^9–12^. Similar to our setup, these studies translationally cool a molecular ion to sub-millikelvin temperatures via the Coulomb interaction with a co-trapped, laser-cooled atomic ion partner. This sympathetic cooling process leaves the ions arranged in an ordered Coulomb crystal, in which they are highly spatially localized and isolated from the environment. These features make laser-cooled Coulomb crystals an ideal platform for multiple forms of spectroscopy^13,14^, in which effectively indefinite trap lifetimes make even very weak transitions accessible^15,16^. Quantum logic techniques in particular remain the gold standard for high-resolution single molecule measurements. In spite of this resolution advantage, these methods are often technically challenging to implement and difficult to apply to arbitrary molecular species. Quantum logic is therefore invaluable for fundamental physics and precision measurement experiments^17,18^, but impractical as a tool for chemical analysis. By adapting non-destructive mass spectrometry methods that are uniquely possible with laser-cooled Coulomb crystals^19^, we are able to record a spectrum via the detection of tagging and de-tagging cycles. This approach is technically simple to implement and is generalizable to a broad class of polyatomic molecules.

## Experimental

**Fig. 1:**
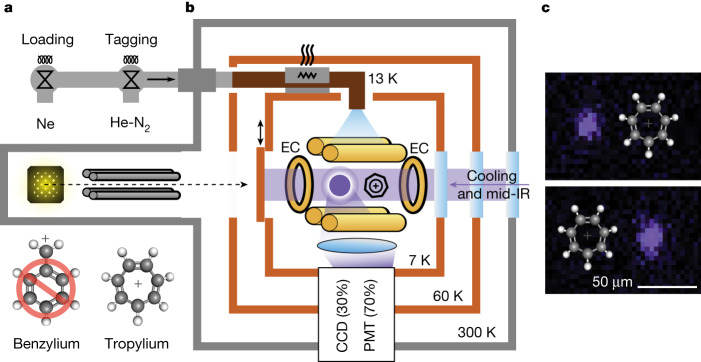
Single molecule tagging setup. **a**, Ions are produced at room temperature and mass filtered before trapping. The competing mass 91 Da isomer benzylium (Bz^+^) is formed, along with Tr^+^, but only Tr^+^ is trapped due to the photodissociation of Bz^+^ by 422 nm laser light^29^. Neon is pulsed into the trapping region during loading to dissipate the kinetic energy of incoming ions via collisions. After loading, a mechanical shutter is closed to separate the cold experimental region from the room temperature ionization region. **b**, Trap details are discussed in the Methods.^88^Sr^+^ and Tr^+^ are co-trapped in a linear quadrupole trap with two endcap (EC) electrodes confining the ions along the trap axis. A 10:1 mixture of He:N_2_ cooled to 13 K is pulsed into the trapping region, tagging the molecular ion with N_2_. An a.c. voltage applied between the endcaps drives secular oscillation, which modulates the ^88^Sr^+^ fluorescence. Lenses below the trap collect fluorescence light and image it onto a CCD camera and PMT. **c**, Two CCD images show ^88^Sr^+^ and Tr^+^ flipping between two possible positions along the trap axis. Tr^+^ is dark to direct observation and is represented by a ball-and-stick model.

^88^Sr^+^ is trapped in a linear Paul trap and Doppler cooled to millikelvin temperatures by driving the 5*p* ^2^P_1/2_ → 5*s* ^2^S_1/2_ transition with red de-tuned 422 nm laser light. About 6 × 10^3^ scattered photons per second are collected and recorded by a charge-coupled device (CCD) camera and a photomultiplier tube (PMT), with 70% going to the PMT for photon correlation measurements (Fig. 1). A single molecular ion is mass selected before being co-trapped with a single ^88^Sr^+^ atomic ion, which sympathetically cools the molecule to millikelvin translational temperatures via the mutual Coulomb interaction between the ions. As for nearly all molecules, Tr^+^ lacks the level structure necessary for efficient laser cooling and rarely scatters photons from the 422 nm cooling light. Hence, the molecular ion is dark to direct observation.

**Fig. 2:**
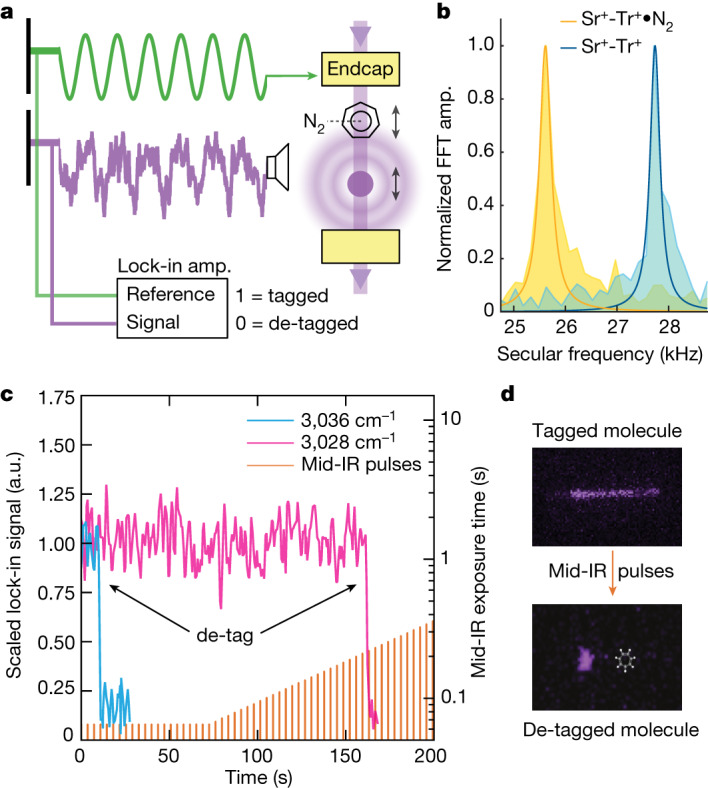
De-tagging detection. **a**, An a.c. drive voltage (green) is resonant with the ^88^Sr^+^–Tr⋅N_2_ secular frequency, causing the ions to oscillate. The resulting Doppler modulation of the ^88^Sr^+^ fluorescence intensity is observed by a PMT (purple). A lock-in amplifier (amp.) compares the PMT signal with the a.c. drive, producing a near-unity voltage when the drive is resonant, and near-zero when not. **b**, The axial secular frequency of a ^88^Sr^+^–Tr^+^ Coulomb crystal decreases by several kHz when Tr^+^ is tagged. The measurement in **a** is done at these two frequencies, and the resulting lock-in signals indicate the tagged state of the molecule. FFT, fast Fourier transform. **c**, A tagged molecule is exposed to a sequence of mid-IR light pulses. The pulses are initially of fixed length, later increasing exponentially in duration. Incident mid-IR photons drive vibrational transitions, which remove the molecule tag. This is observed as a discontinuous drop in the lock-in amplifier signal. Fast (blue) and slow (pink) de-tagging events are shown. When a de-tagging event is detected, the total exposure time from the mid-IR pulse train is summed and inverted to produce a de-tagging rate. a.u., arbitrary units. **d**, CCD camera images of a Coulomb crystal with a tagged molecule driven to oscillate, which ceases oscillation after de-tagging.

The mass of the molecular ion is determined non-destructively by observing the photons scattered by ^88^Sr^+^. The Coulomb crystallized molecular ion–atomic ion pair forms a coupled oscillator confined within the pseudo-harmonic potential of the trap, with a characteristic secular frequency that depends on the mass of both ions. We drive the ion pair to oscillate at the secular frequency, which modulates the ^88^Sr^+^ fluorescence intensity at the same frequency via the Doppler effect. This modulation can be directly observed with a PMT (Fig. 2a).

For the two-ion Coulomb crystals studied here, there is a simple analytical relationship between axial oscillation frequency of the ions and their total mass^20^. The mass of the atomic ion is already known precisely, however, so that a measurement of the axial secular frequency of the crystal directly reveals the mass of the molecular ion. We use a novel chirped voltage pulse method to initially measure the axial frequency of the mixed Coulomb crystal, both with and without a tag attached to the molecule (Methods). Once measured, we detect the presence and absence of tags by driving ion motion at the ^88^Sr^+^–Tr⋅N_2_ resonant frequency. A lock-in amplifier compares the drive frequencies with the observed ^88^Sr^+^ fluorescence modulation and outputs a signal proportional to the fluorescence correlation. This process is illustrated in Fig. 2. Changes in the tag state of the molecule are therefore observed as discontinuous changes in the lock-in signal amplitude.

Nitrogen is our tag species of choice, as it readily tags most molecules and remains attached with a long lifetime, enabling us to observe weak transitions through slow de-tagging events. Molecular ions are tagged with N_2_ using a 10:1 mixture of He:N_2_. Short pulses of this gas mixture flow through a cryogenic heat exchanger, which cools it to 13 K before it enters the trapping region. Helium efficiently removes kinetic energy from the system during ternary molecule–He–N_2_ collisions, leaving N_2_ weakly bound to the molecule. In the case of Tr^+^, the lifetime of this complex is several hours in the absence of mid-infrared (IR) laser light. This leads to a near-zero background de-tagging rate on the timescale of our experiment, enabling us to observe very weak spectral features that are invisible to most other methods.

**Fig. 3:**
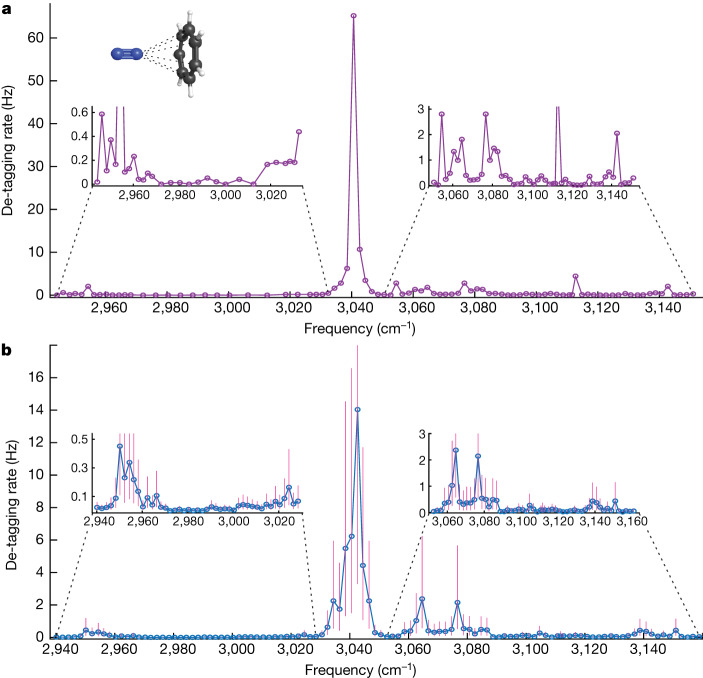
Single molecule spectrum. **a**, The infrared spectrum of a single Tr^+^ molecule in the C–H stretching region. The strong transition at 3,040 cm^−1^ is consistent with previously reported values for N_2_-tagged Tr^+^ (ref. ^4^), which assign this transition as an asymmetric C–H stretch. Weak transitions, invisible to most other action techniques, are shown in the insets. We broadly attribute these features to combination bands. **b**, A composite of eight single molecule measurements. Three de-tagging measurements were made every 2 cm^−1^. Maximum likelihood estimates of the de-tagging time constant are calculated at each point, as described in the Methods, with vertical bars indicating 95% confidence intervals for these values. The primary peak shifts slightly to 3,042 cm^−1^ with these additional measurements. The confidence interval on the primary peak extends to 39 Hz. A total of eight Tr^+^ molecules were used to measure all plotted points, as molecules were periodically lost due to reactive collisions with background gas.

Molecules are de-tagged with mid-IR light from a commercial optical parametric oscillator (OPO). The OPO output has a spectral linewidth of 6 cm^−1^ over the region of interest and is co-aligned with light from the cooling lasers along the axis of the ion trap. As shown in Fig. 2a, a Coulomb crystal with a single tagged Tr^+^ is driven to oscillate at its secular frequency by a sinusoidal voltage applied to a trap endcap electrode. This drive signal is sent to a lock-in amplifier, where it is compared to the fluorescence signal observed by the PMT. The frequencies of the two signals are initially equal, thus producing a high lock-in output signal. A sequence of mid-IR pulses of varying length are sent to the trap while the secular motion is being driven. Resonant de-tagging of the molecule causes the secular frequency of the ions to jump discontinuously (Fig. 2b), eliminating the correlation between the applied drive voltage and the observed fluorescence modulation (Fig. 2c). The observed lock-in signal therefore drops to near-zero, indicating that a de-tagging event has occurred. The total duration of all mid-IR pulses up to the point of de-tagging is then recorded, providing a de-tagging time and corresponding rate. The molecular ion is then re-tagged for further measurements at different mid-IR laser frequencies. Near resonance, the mid-IR light de-tags molecular ions on a timescale of tens of milliseconds. Off resonance, no de-tagging events occur within 90 seconds, giving a baseline >90 seconds. This wide dynamic range makes weaker transitions with lifetimes of many seconds easily observable. The total time required to acquire a data point in a spectrum varies with the de-tagging time, but, on average, it takes approximately two minutes to prepare a tagged molecule and to subsequently de-tag it. Although a molecule can be recycled for many tag–de-tag cycles, it is occasionally lost to reactions with background contaminants. Under favourable circumstances, as shown in Fig. 3a, we can obtain a spectrum from a single molecule, although in our follow-up work with other molecules we typically found that between one and five molecules are needed to obtain a clear spectrum^21^. Although the data acquisition time for our method is much longer than in traditional tagging spectroscopy, the innate purity of our single molecule samples results in simpler observed spectra.

## Single molecule spectrum

The vibrational spectrum of a single Tr^+^ molecule in the C–H stretching region is shown in Fig. 3a, spanning 2,944–3,150 cm^−1^. These single molecule data are consistent with a composite spectrum taken from eight individual Tr^+^ ions, as shown in Fig. 3b. A maximum likelihood estimate of the de-tagging time constant at every frequency step is calculated from the three de-tagging observations. The de-tagging rates reported in Fig. 3 are the inverse of these calculated time constants. The vertical bars in Fig. 3b are 95% confidence intervals derived from the de-tagging probability distribution at each frequency step, which is centred on the estimated most likely time constant. Single molecule measurements from eight total molecular ions were used for this averaged spectrum, as the molecules were occasionally lost from the trap. These losses are attributed to reactive collisions with residual background gases, such as O_2_.

The dominant feature in this spectrum at 3,042 cm^−1^ is consistent with previous tagging experiments^4^, which assigned this transition as the lone IR-active, asymmetric C–H stretching mode of Tr^+^. A single transition at 3,074 cm^−1^ was also reported, but the enhanced resolution of our method compared to conventional tagging spectroscopy enables us to resolve a previously unseen splitting of this peak into two features at 3,065 and 3,077 cm^−1^. Numerous studies of Tr^+^ have observed this band^22^, but it has yet to be assigned. In addition to these primary peaks, we observe weak transitions at 2,952 and 3,140 cm^−1^. These peaks lie below the noise floor of other action spectroscopy methods and have therefore not been reported previously. Theoretical work to aid in the detailed assignment of all four weak bands is ongoing, but these are probably attributable to weak combination or overtone transitions.

Although our method measures the absorption spectrum of the molecular ion–tag van der Waals complex, such as Tr^+^⋅N_2_, this spectrum is a very close analogue to that of the bare molecular ion. Selection rules and transition frequencies are slightly perturbed by the presence of a tag, but typical line shifts are of the order of a few cm^−1^ (ref. ^23^). Proposed^15^ and demonstrated^24^ methods to study bare molecular ions are compatible with the single molecule technique demonstrated here, and, in principle, could leverage the highly controlled environment of the Coulomb crystal to yield very high resolution, rotationally resolved single molecule spectra.

The linewidths observed for transitions in Fig. 3 are limited both by the spectral profile of our OPO light source as well as by the natural timescale for the de-tagging process. Although this timescale is different for each molecule, it is predicted to range anywhere from 0.1 to 100 ps (ref. ^25^), giving a natural frequency resolution limit of the order of 10 GHz–10 THz. Our observed noise level is dominated by sampling error, which is proportional to the de-tagging rate and is reduced by repeated measurements. We find that three repetitions at each wavelength sampled are sufficient to reduce our sampling error, to identify repeatable spectral features. As the tagged lifetime for Tr⋅N_2_ is several hours in the absence of mid-IR light, and >90 seconds off resonance, we are able to measure de-tagging events up to 90 seconds with a near-zero background.

Tr^+^ has been the subject of exhaustive study for over a century, largely owing to the long-held belief that this unique aromatic cation contributes to the *m* = 91 Da component of alkylbenzene mass spectra^26,27^. Tagging spectroscopy has recently confirmed the presence of Tr^+^ in such fragmentation processes^4,28^, along with isomeric benzylium (Bz^+^; Fig. 1). The two isomers often form at similar rates and thus both contribute to observed spectra, complicating the assignment process. Although our single molecule technique is uniquely suited to distinguish between these competing isomers through sequential measurements, this process proves unnecessary in our system. Bz^+^ is known to readily dissociate when exposed to 422 nm light^29^, which is necessarily present in our system for laser cooling. Tr^+^ is therefore the only mass 91 Da isomer that we observe, which is confirmed by the absence of known Bz^+^ transitions at 2,997 or 3,116 cm^−1^ in our spectra^4^. Additionally, any isomerization of trapped Tr^+^ to Bz^+^ would lead to rapid photodissociation and spontaneous loss of the molecule, which we do not observe. Bz^+^ studies could easily be realized with the choice of a different atomic species for laser cooling.

The perturbations caused by the van der Waals-adhered tag do not prevent identification of the molecular ion through the measurement of vibrational transitions. In general, the infrared absorption spectrum as measured here is insufficient to determine the structure of a previously unknown species, but is sufficient to distinguish different species and to definitively identify a compound if its spectrum is previously known. Such identification could prove useful in the analysis of rare samples. For example, molecules from the moons of Saturn have been characterized in situ previously via mass spectrometry^6^, with organic molecular ions, such as C_7_H_7_^+^, having been identified. Definitive identification of the structures of these molecules is beyond the state of the art for such tools however, making an adaptation of our single molecule spectroscopic method an attractive alternative for such rare samples. Competing isomers of C_7_H_7_^+^ could be resolved given the library of spectra already obtained for the two isomers. Extension to more complex mixtures would require a library of spectral data for the expected molecules at each mass. In principle, a mature version of our single molecule method would enable the deconvolution of complex mixtures with no confusion from overlapping spectra. We also expect our single molecule method could find application in characterizing mixed-species products produced in cold and astrochemically relevant chemical reactions^30,31^, without the need for previous purification. As our spectral measurement is non-destructive to the analyte molecule, the spectra could be used to identify both charged reactants and products. In this case, ab initio calculations would guide the assignment by narrowing the list of likely reaction products on the basis of energetics. Although measuring the C–H stretch as we have shown here could provide identification for sufficiently distinct candidate molecules, our method is easily extended to the fingerprint region (between 400 and 1,500 cm^−1^), where low-energy C–H bending modes provide molecule-specific transitions. Experiments are ongoing to demonstrate this technique on other molecular ion species and study the product distribution of a single molecule photofragmentation reaction.

## Methods

### Trap details and chirped excitation mass measurement

**Extended Data Fig. 1:**
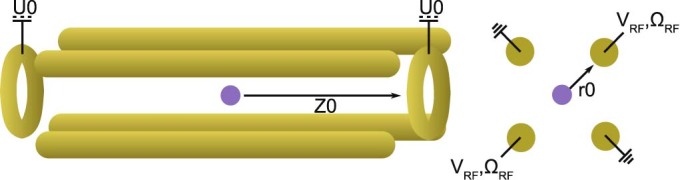
Trap diagram. A sketch of the trap showing the side view (left) and cross-sectional view (right) for relevant parameters described in Extended Data Table 1.

This experiment takes place in a linear Paul trap (Extended Data Fig. 1) mounted to a closed-cycle helium cryocooler, which enables both cryo-pumping and buffer gas tagging at cryogenic temperatures. Relevant trap parameters are listed in Extended Data Table 1. Oscillating radiofrequency voltages of amplitude *V* and angular frequency Ω, applied to two opposing rods create a stable trapping region along the radial (*r*) direction of the trap for particles with charge *Q* and mass *m* when the Matthieu parameter $q=\frac{2QV}{m{r}^{2}{\varOmega }^{2}} < 0.9$. In practice, the trap is operated at 0.2 < *q*_88_ < 0.4 to stably confine a wide range of masses. An oscillating voltage applied to a trap electrode at the mass-dependent radial secular frequency ${\omega }_{r}\approx \frac{QV}{\sqrt{2}m{r}^{2}\varOmega }$ is used for mass-selective ejection of unwanted species from the trap, for example after a reactive collision with background gas.

The coupling between the ions along the axial (*Z*) direction of the trap, arising from their mutual Coulomb interaction, is strong compared to the static field (*U*_0_) applied to the ring-shaped endcap electrodes. A two-ion Coulomb crystal has two modes of oscillation along the axial direction: a centre of mass (*ω*_*z*,COM_) and a breathing (*ω*_*z*,BM_) mode. The oscillation frequencies of these modes depend on the mass of both ions, *μ* = *m*_Sr_/*m*_molecule_, as shown in equations (1) and (2) (refs. ^32–34^). Both modes are observed, but *ω*_*z*,COM_ is easier to drive and detect as it does not require a field gradient to excite. Hence, this is the mode used for the non-destructive mass detection.

1 $${\omega }_{z,{\rm{COM}}}^{2}=[(1+\mu )-\sqrt{1-\mu +{\mu }^{2}}]{\omega }_{z,88}^{2}$$

2 $${\omega }_{z,{\rm{BM}}}^{2}=[(1+\mu )+\sqrt{1-\mu +{\mu }^{2}}]{\omega }_{z,88}^{2}$$

In a typical experimental cycle, a single ^88^Sr^+^ is trapped and the secular frequency is measured to serve as a calibration. A single molecule is mass selected before co-trapping with ^88^Sr^+^, and the mass is verified by an additional secular frequency measurement. Although we use only two total ions for this spectrum, the number of ^88^Sr^+^ cooling partners could easily be increased to improve the sympathetic cooling efficiency for the molecule. This may be advantageous for molecules with a large mass disparity compared to ^88^Sr^+^, in which the sympathetic cooling efficiency with a single cooling partner is poor.

**Extended Data Fig. 2:**
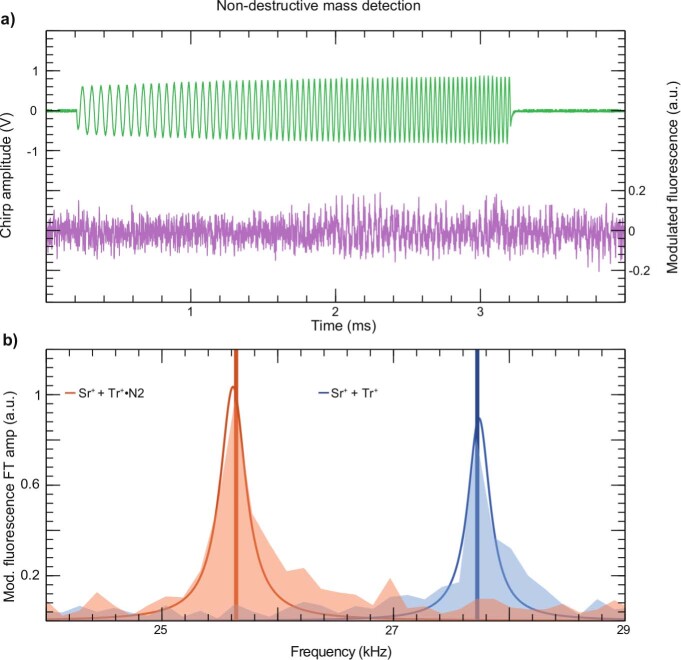
Non-destructive mass detection. **a**, An example chirp waveform applied to the ion pair (green) and the resulting modulated fluorescence (purple). Ion oscillation can be seen to begin around the 2 ms point. **b**, The modulated fluorescence signal is Fourier transformed (shaded) and fit with a Lorentzian function (line profile) to extract a centre frequency used for mass determination. Vertical bars indicate the calculated secular frequency from the measured single ^88^Sr^+^ secular frequency (not shown).

We directly measure the axial secular frequency of a molecule–^88^Sr^+^ pair through a chirped-ringdown measurement^19^. ‘Chirp’ refers to a pulsed cosine waveform in which the frequency increases linearly in time over the duration of the pulse. Such a waveform in the time domain corresponds to an approximate square wave in the frequency domain. The chirp is tuned to sweep across the expected secular frequencies for a Coulomb crystal in our trap, and is applied to a trap endcap to drive axial motion. When the chirp frequency approaches the axial secular frequency of the ion pair, the ions oscillate in phase with a large amplitude. This oscillation Doppler shifts the ^88^Sr^+^ ion in and out of resonance with the 422 nm cooling light, modulating its resulting fluorescence intensity at the secular frequency of the ions. Although the chirp frequency quickly sweeps past the secular frequency of the ions, they continue to oscillate with decaying amplitude, producing a ‘ringdown’ in the observed fluorescence modulation (Extended Data Fig. 2). This decay timescale is the result of damping caused by the ^88^Sr^+^ laser cooling process, and the strength of this damping depends on the 422 nm laser detuning and intensity relative to the 5*p* ^2^P_1/2_→5*s* ^2^S_1/2_ cooling transition. We typically operate 60 MHz red de-tuned from resonance and slightly below the saturation intensity of the transition to maximize the modulated fluorescence signal.

The chirp amplitude applied to the trap has a typical amplitude of the order of 10 mV peak to peak, sweeping from 25 to 35 kHz over a span of 3 ms. A 2 ms ringdown time after the chirp enables the Doppler-modulated fluorescence to decay as the ions cool back to equilibrium. This cycle is then repeated many times to enable signal averaging. During the chirp cycle, fluorescence is collected by a PMT, the output of which is averaged on an oscilloscope triggered on the start of the chirp waveform. About 2,000 averages of the fluorescence collected over the chirp cycle are required for a clear fluorescence modulation signal and these typically take about 30 s to acquire. The fluorescence signal is Fourier transformed to extract a secular frequency, as shown in Extended Data Fig. 2, to verify the mass of the analyte molecule and the presence of the N_2_ tag. Chirp parameters and number of averages are changed for other molecules with different expected secular frequencies and potentially slower sympathetic cooling rates in the case of a large size mismatch. Using this method, we observe masses ranging from about 50 to 260 Da.

### Individual molecule datasets

**Extended Data Fig. 3:**
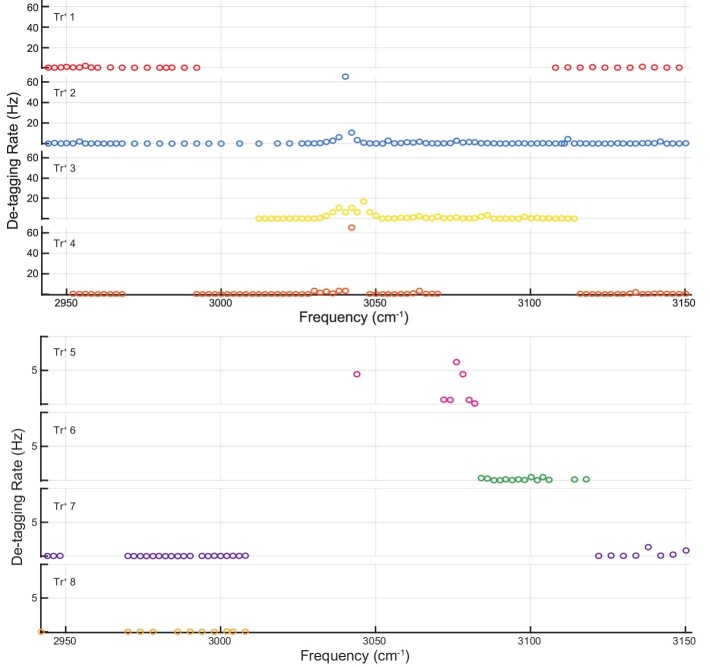
Individual molecular spectra. The data points recorded for each of the eight individual molecules used in the averaged spectrum in the main text. The majority of points in the spectrum were recorded with the first four molecules, and the remainder were used primarily to observe background points.

The spectrum plotted in Fig. 3b in the main text is an average of eight different single molecule spectra, as shown in Extended Data Fig. 3. Summing across all Tr^+^ molecules that were interrogated, a total of three de-tagging measurements were observed at every frequency step in the spectrum. More than one Tr^+^ was needed to acquire this full spectrum, as we suspect background O_2_ periodically reacted with the molecules during the investigation. The occasional formation of SrO^+^ indicates the presence of reactive background collisions. The spectral data from each of these eight molecules are plotted below in the order in which they were recorded.

We note that each time data acquisition commenced with a new Tr^+^ molecule we verified the presence of the strong transition near 3,042 cm^−1^ to confirm that it was a Tr^+^ rather than a Bz^+^ isomer. Additionally, we calibrated the frequency axis of our spectra using two different references. We recorded absorption spectra with our mid-IR source for solid polystyrene, as well as for ammonia vapour. Known peak positions for both species are provided by NIST, and we fit a simple linear calibration curve to this reference data and apply this linear transformation to our observed frequency data.

**Extended Data Fig. 4:**
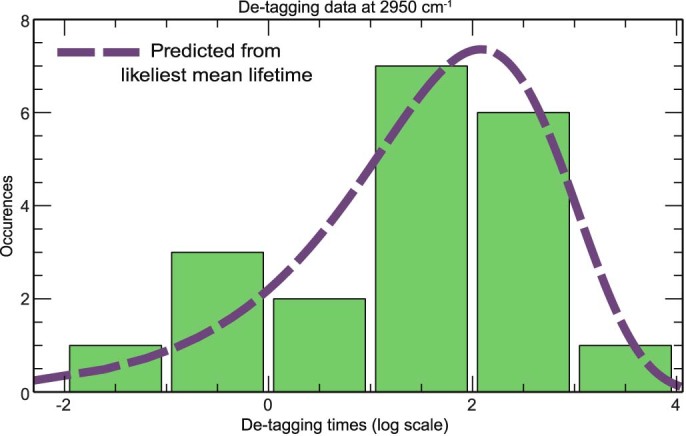
Single mid-IR frequency de-tagging histogram. A sample histogram of 20 lifetime measurements on the weak spectral feature at 2,950 cm^−1^ shows consistency with the model of the exponential distribution.

Each mid-IR frequency data point shows a distribution of de-tagging rates, as shown in Extended Data Fig. 4. The full set of de-tagging data shown in Extended Data Fig. 3 was compiled into the spectrum reported in the main text.

### Computational details

Preliminary computational results indicate that there may be multiple energetically competitive attachment points for N_2_ on Tr^+^. Two degenerate ‘face’ sites have been identified, for which N_2_ is orthogonal to the Tr^+^ plane, and seven degenerate ‘side’ sites, for which N_2_ lays in the Tr^+^ plane. As previously noted, we see strong experimental evidence that only one such tagging site is ever occupied during this experiment, implying that one of the above possibilities must be much more energetically favourable than the others. The de-tagging spectrum that we observe is affected by the exact tagging site, however, as the various geometries have various levels of symmetry. An effort was therefore made to computationally distinguish the two classes of tagging sites to try to identify the tagging site that we observe experimentally.

Geometry optimization of the tagged structures was performed using several different methods: B3LYP-D3, M06-2X and MP2, all using Dunning’s aug-cc-pVTZ basis sets^35^ in the Gaussian 16 software package^36^. Results were, however, inconclusive (Extended Data Table 2, with density functional theory methods finding a higher binding energy for side-tagged species, and MP2 methods finding higher binding energies for face-tagged sites. Importantly, all of the calculated binding energies were well below the IR photon energies used in this work, so that all reported de-tagging events arise from single-photon processes. However, the inconclusive results indicate the need for more expensive coupled-cluster calculations for this system.

### Notes on data analysis

We measure the mid-IR exposure time required for a de-tagging event, *T*, and model it as a random variable following an exponential distribution. Once a tagged molecule has absorbed a resonant photon, de-tagging occurs at the picosecond level. This timescale is much shorter than the time between individual pulses of our mid-IR pulsed laser, which has a repetition rate of 150 kHz. At a given mid-IR laser frequency, *ω*_L_, the probability that a tagged molecule de-tags during a laser pulse is a constant. As a result, the probability that the de-tagging event happens right after a sequence of pulses is exponential in the number of pulses. As the total exposure time before de-tagging is proportional to the total number of exposure pulses, *T* is an exponentially distributed random variable. The probability density function (*p*) of the distribution is characterized by the wavelength-dependent exponential time constant *τ*(*ω*_L_), which can be expressed as

3 $$p(T,{\omega }_{{\rm{L}}})=\frac{1}{\tau ({\omega }_{{\rm{L}}})}{{\rm{e}}}^{\frac{-T}{\tau ({\omega }_{{\rm{L}}})}}$$

On exposure to mid-IR light, de-tagging occurs on a timescale faster than the detection time for our mass measurement methods. To extract a meaningful rate, the mid-IR laser is gated via a mechanical shutter into a sequence of exposures. The 2.5 s intervals between exposures enable the lock-in signal to respond to the mass change to the molecule caused by a de-tagging event. The first few of these exposures last for tens of milliseconds each, enabling us to capture fast de-tagging events. After a set number of constant fast exposures, the exposure duration is increased exponentially to observe slow de-tagging events within a reasonable experimental timescale.

When a de-tagging event is observed, the start time, *T*_s_, is defined as the total time the molecule was exposed to the mid-IR radiation before the final de-tagging pulse. The final time, *T*_f_, is taken as *T*_s_ plus the length of the final pulse. The de-tagging event occurs sometime between these two, *T*_s_ and *T*_f_. Given the probability distribution in equation (3), the probability (*P*) of observing a de-tagging event between *T*_s_ and *T*_f_ is

4 $$P({T}_{{\rm{s}}},{T}_{{\rm{f}}}| \tau ({\omega }_{{\rm{L}}}))={{\rm{e}}}^{-\frac{{T}_{{\rm{s}}}}{\tau ({\omega }_{{\rm{L}}})}}-{{\rm{e}}}^{-\frac{{T}_{{\rm{f}}}}{\tau ({\omega }_{{\rm{L}}})}}$$

For three independent measurements per point, this becomes

5 $$P({T}_{i}| \tau ({\omega }_{{\rm{L}}}))=\mathop{\prod }\limits_{i=1}^{3}\left[{{\rm{e}}}^{-\frac{{T}_{{\rm{s}},i}}{\tau ({\omega }_{{\rm{L}}})}}-{{\rm{e}}}^{-\frac{{T}_{{\rm{f}},i}}{\tau ({\omega }_{{\rm{L}}})}}\right]$$

Assuming that the previous probability of ln(*τ*(*ω*_L_)) is uniformly distributed, the maximum a posteriori estimation or the maximum likelihood estimation of the distribution parameter *τ*(*ω*_L_) is achieved by maximizing the likelihood function *L*_3_(*τ*(*ω*_L_)∣*T*_*i*_) = *P*(*T*_*i*_∣*τ*(*ω*_L_)) with respect to *τ*(*ω*_L_). We maximize this function numerically to determine the most likely value for *τ*(*ω*_L_). We also determine a 95% confidence level numerically on the basis of the distribution of the likelihood functions. The density of the confidence level is proportional to the posterior probability density, and we have

6 $${\rm{CL}}({{\rm{CI}}}_{{\rm{\min }}} < \tau  < {{\rm{CI}}}_{{\rm{\max }}})\equiv \frac{{\int }_{\tau ={{\rm{CI}}}_{{\rm{\min }}}}^{\tau ={{\rm{CI}}}_{{\rm{\max }}}}{L}_{3}(\tau | {T}_{i})\,{\rm{dln}}(\tau )}{{\int }_{\tau =0}^{\tau =+\infty }{L}_{3}(\tau | {T}_{i})\,{\rm{dln}}(\tau )}$$

We choose the interval in which the likelihood function *L*_3_(*τ*(*ω*_L_)∣*T*_*i*_) > e^−2^*L*_3,max_ as our confidence interval, and verify that this corresponds to a confidence level of ≳95%.

From the point of view of random sampling, our method of data analysis can be well justified when ignoring the slight broadening and distortion of the likelihood function due to the uncertainty caused by the finite exposure time from our shuttered laser source. Assuming the observations *T*_*i*_ constitute *N* random samples subjected to an exponential distribution with the time constant *τ*_0_, and d*T*_*i*_ ≡ *T*_f,*i*_ − *T*_s,*i*_ ≪ *τ*_0_ for all *i*, we have,

7 $${L}_{N}(\tau | {T}_{i})=\mathop{\prod }\limits_{i=1}^{N}\left[\frac{{\rm{d}}{T}_{i}}{\tau }{{\rm{e}}}^{-\frac{{T}_{i}}{\tau }}\right]$$

The likelihood function is maximum when $\tau =\frac{{\sum }_{i=1}^{N}{T}_{i}}{N}$, which gives an unbiased estimator of *τ*_0_. Notably, the likelihood function *L*_*N*_ can be normalized by a factor ${\left(\frac{{\sum }_{i=1}^{N}{T}_{i}}{N}\right)}^{N}\cdot \frac{1}{{\prod }_{i=1}^{N}{\rm{d}}{T}_{i}}$ to give a function only of the number of samples *N* and the relative magnitude of *τ* and $\frac{{\sum }_{i=1}^{N}{T}_{i}}{N}$. This indicates that for a given *N*, *L*_*N*_ is always proportional to a fixed function ${\rm{ln}}\,\left(\frac{N\tau }{{\sum }_{i=1}^{N}{T}_{i}}\right)$. This differs from cases for which random variables are normally distributed. For any *v* > *u* > 0, if we choose the confidence interval to be ${\rm{C}}{\rm{I}}=\left(u\frac{\sum {T}_{i}}{N},v\frac{\sum {T}_{i}}{N}\right)$, the confidence level is a constant for any set of *T*_*i*_ and equal to

8 $$\begin{array}{c}{\rm{C}}{\rm{L}}\,\left(u\frac{\sum {T}_{i}}{N} < \tau  < v\frac{\sum {T}_{i}}{N}\right)\,\equiv \,\frac{{\int }_{\tau ={{\rm{C}}{\rm{I}}}_{min}}^{\tau ={{\rm{C}}{\rm{I}}}_{max}}{L}_{N}(\tau |{T}_{i})\,{\rm{d}}{\rm{l}}{\rm{n}}(\tau )}{{\int }_{\tau =0}^{\tau =+{\rm{\infty }}}{L}_{N}(\tau |{T}_{i})\,{\rm{d}}{\rm{l}}{\rm{n}}(\tau )}\\ \,\,\,\,\,\,=\,P\,\left(\frac{{\tau }^{{\prime} }}{v} < \frac{\sum {T}_{i}^{{\prime} }}{N} < \frac{{\tau }^{{\prime} }}{u}|{\tau }^{{\prime} }\right)\end{array}$$

The right-hand side of equation (8) is the probability that the arithmetic mean of *N* random samples from an exponential distribution with time constant *τ*′ falls within the interval $\left(\frac{{\tau }^{{\prime} }}{v},\frac{{\tau }^{{\prime} }}{u}\right)$. It is a constant for any *τ*′ > 0. If we consider a special case in which *τ*′ = *τ*_0_, the right-hand side of equation (8) is also the probability that the confidence interval determined for *N* samples includes the true value *τ*_0_. Hence, our method gives a reasonable estimation of the time constant, confidence interval and the confidence level.

Two wavelengths were chosen to test this model of the distribution, with a sample histogram at 2,950 cm^−1^ shown in Extended Data Fig. 4. For this test, we measured 20 de-tagging times for Tr^+^ irradiated with single frequency laser light at 2,950 cm^−1^. We observe a uniform distribution, which is consistent with the exponential model described above. Additionally, we see no evidence of bimodality in this distribution, which might arise if the N_2_ attaches to Tr^+^ at different sites during successive measurements. Such effects from multiple tagging sites could be readily observed in other systems, however, by measuring the de-tagging time distribution for observed transitions at a single laser frequency.

## Online content

Any methods, additional references, Nature Portfolio reporting summaries, source data, extended data, supplementary information, acknowledgements, peer review information; details of author contributions and competing interests; and statements of data and code availability are available at 10.1038/s41586-023-06351-7.

## Figures and Tables

**Extended Data Table 1:**
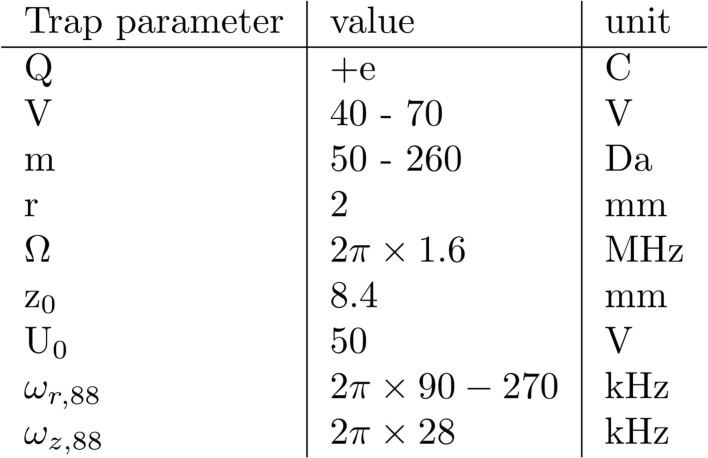
Trap parameters

Typical trap parameters for our cryogenic linear Paul trap used in this experiment. Extended Data Fig. 1 displays a trap diagram.

**Extended Data Table 2:**
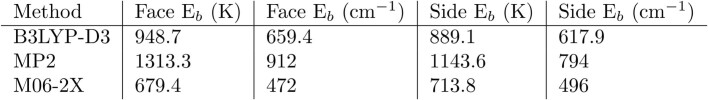
Computational results

A table of computed binding energies in K and cm^−1^.

## Data Availability

All data acquired are presented with the paper. Raw data are available from the corresponding author on request.

## References

[1] Wolk AB, Leavitt CM, Garand E, Johnson MA (2013). Cryogenic ion chemistry and spectroscopy. Acc. Chem. Res..

[2] Pereverzev A, Roithová J (2022). Experimental techniques and terminology in gas-phase ion spectroscopy. J. Mass Spectrom..

[3] Campbell EK, Holz M, Gerlich D, Maier JP (2015). Laboratory confirmation of C_60_^+^ as the carrier of two diffuse interstellar bands. Nature.

[4] Wagner JP, McDonald II DC, Duncan MA (2018). Mid-infrared spectroscopy of C_7_H_7_^+^ isomers in the gas phase: benzylium and tropylium. J. Phys. Chem. Lett..

[5] Naraoka H (2023). Soluble organic molecules in samples of the carbonaceous asteroid (162173) Ryugu. Science.

[6] Waite Jr JH (2006). Cassini ion and neutral mass spectrometer: Enceladus plume composition and structure. Science.

[7] Moerner WE, Kador L (1989). Optical detection and spectroscopy of single molecules in a solid. Phys. Rev. Lett..

[8] Braeken E (2009). Single molecule probing of the local segmental relaxation dynamics in polymer above the glass transition temperature. J. Am. Chem. Soc..

[9] A. K. Hansen MAS, Staanum PF, Drewsen M (2012). Single-ion recycling reactions. Angew. Chem. Int. Edn.

[10] Wolf F (2016). Non-destructive state detection for quantum logic spectroscopy of molecular ions. Nature.

[11] Sinhal M, Meir Z, Najafian K, Hegi G, Willitsch S (2020). Quantum-nondemolition state detection and spectroscopy of single trapped molecules. Science.

[12] Sinhal, M. & Willitsch, S. in *Photonic Quantum Technologies* (ed. Benyoucef, M.) Ch. 13 (John Wiley & Sons, 2023).

[13] Calvin AT, Brown KR (2018). Spectroscopy of molecular ions in Coulomb crystals. J. Phys. Chem. Lett..

[14] Willitsch S (2012). Coulomb-crystallised molecular ions in traps: methods, applications, prospects. Int. Rev. Phys. Chem..

[15] Khanyile NB, Shu G, Brown KR (2015). Observation of vibrational overtones by single-molecule resonant photodissociation. Nat. Commun..

[16] Germann M, Tong X, Willitsch S (2014). Observation of electric-dipole-forbidden infrared transitions in cold molecular ions. Nat. Phys..

[17] Chou C-W (2017). Preparation and coherent manipulation of pure quantum states of a single molecular ion. Nature.

[18] Alighanbari S, Giri GS, Constantin FL, Korobov VI, Schiller S (2020). Precise test of quantum electrodynamics and determination of fundamental constants with HD^+^ ions. Nature.

[19] Sheridan K, Keller M (2011). Weighing of trapped ion crystals and its applications. New J. Phys..

[20] Morigi G, Walther H (2001). Two-species Coulomb chains for quantum information. Eur. Phys. J. D.

[21] Eierman, S. et al. A cryogenic ion trap for single molecule vibrational spectroscopy. *Rev. Sci. Instrum.* (in the press). 10.1063/5.0147695 37477553

[22] Fateley WG, Lippincott ER (1957). Vibrational spectrum and structure of the tropylium ion. J. Chem. Phys..

[23] Johnson CJ (2014). Communication: He-tagged vibrational spectra of the SarGlyH^+^ and H^+^(H_2_O)_2,3_ ions: quantifying tag effects in cryogenic ion vibrational predissociation (CIVP) spectroscopy. J. Chem. Phys..

[24] Schmid PC, Asvany O, Salomon T, Thorwirth S, Schlemmer S (2022). Leak-out spectroscopy, a universal method of action spectroscopy in cold ion traps. J. Phys. Chem. A.

[25] Puttkamer K, Dübal HR, Quack M (1983). Time-dependent processes in polyatomic molecules during and after intense infrared irradiation. Faraday Discuss..

[26] Merling G (1891). Ueber Tropin. Ber. Dtsch. Chem. Ges..

[27] Rylander PN, Meyereson S, Grubb HM (1957). Organic ions in the gas phase. II. The tropylium ion. J. Am. Chem. Soc..

[28] Jusko P, Simon A, Banhatti S, Brünken S, Joblin C (2018). Direct evidence of the benzylium and tropylium cations as the two long-lived isomers of C_7_H_7_^+^. ChemPhysChem.

[29] Fèraud G, Dedonder-Lardeux C, Soorkia S, Jouvet C (2014). Photo-fragmentation spectroscopy of benzylium and 1-phenylethyl cations. J. Chem. Phys..

[30] Heazlewood, B. R. & Lewandowski, H. J. in *Emerging Trends in Chemical Applications of Lasers* (eds Berman, M. R. et al.) ACS Symposium Series, Vol. 1398, pp. 389–410 (American Chemical Society, 2021).

[31] Krohn OA, Catani KJ, Lewandowski HJ (2022). Formation of astrochemically relevant molecular ions: reaction of translationally cold CCl^+^ with benzene in a linear ion trap. Phys. Rev. A.

[32] Kielpinski D (2000). Sympathetic cooling of trapped ions for quantum logic. Phys. Rev. A.

[33] Rajagopal V, Marler JP, Kokish MG, Odom BC (2016). Trapped ion chain thermometry and mass spectrometry through imaging. Eur. J. Mass Spectrom..

[34] Fan M (2021). Optical mass spectrometry of cold RaOH^+^ and RaOCH_3_^+^. Phys. Rev. Lett..

[35] Dunning Jr TH (1989). Gaussian basis sets for use in correlated molecular calculations. I. The atoms boron through neon and hydrogen. J. Chem. Phys..

[36] Frisch, M.J. et al. Gaussian 16 Revision C.01 (Gaussian, 2016).

